# National outpatient antibiotic prescriptions in Cyprus, 2020–2022

**DOI:** 10.1017/ash.2025.10079

**Published:** 2025-10-15

**Authors:** Eirini Mitsoura, Ioannis Kopsidas, Eleni Kourkouni, Annalisa Quattrocchi, George Samoutis, Panagiotis Petrou, Andreas Sarantopoulos, George Papazisis, Nikolaos Raikos, Zoi Dorothea Pana

**Affiliations:** 1 Medical School, University of Nicosia (UNIC), Nicosia, Cyprus; 2 Medical School, Aristotle University Thessaloniki (AUTH), Thessaloniki, Greece; 3 Center for Clinical Epidemiology and Outcomes Research (CLEO), Athens, Greece; 4 Health Insurance Organization Cyprus (HIO), Nicosia, Cyprus; 5 Medical School, European University Cyprus, Nicosia, Cyprus

## Abstract

**Background:**

The misuse of antibiotics and the consequential rise in antimicrobial resistance (AMR) represent a significant global health concern. Cyprus currently reports to ESAC-Net the total consumption of antimicrobials, without distinguishing between hospital- and community-based antibiotic use. As a result, these data can only provide generalized insights into antimicrobial trends in the country.

**Aim:**

To evaluate antibiotic prescribing patterns in national outpatient services in Cyprus.

**Material and methods:**

We retrospectively analyzed the national community antibiotic prescriptions in Cyprus for the period 2020 – 2022. Data on community antimicrobial prescription were extracted from the National Insurance Organization (HIO) database. Orally administered dispensed antibiotics were categorized according to the WHO Anatomical Therapeutic Chemical (ATC) classification and by the WHO’s AWaRe (Access, Watch, Reserve) classification of antibiotics. Antibiotic prescriptions were calculated in both packages consumed and per 1000 beneficiaries, overall, by year, age groups, and medical specialty of prescribers.

**Results:**

During the period of 2020 – 2022, in total 1,458,723 antibacterial prescriptions for systemic use were ordered in Cyprus for outpatients. The annual rates of prescriptions per 1,000 beneficiaries demonstrated an increasing trend from 535.7 in 2020 to 653 in 2022, respectively. A decrease by 3.1% in the prescription of WHO “Access” antibiotics was observed, reaching 42.3% in 2022, which is much lower than the WHO’s goal of 60% and the EU’s goal of 70% for “Access” antibiotic consumption.

**Conclusions:**

Between 2020 and 2022, antibiotic prescriptions in the community of Cyprus showed an overall increase, accompanied by a declining trend in the proportion of “Access” antibiotics prescribed.

## Introduction

The misuse of antibiotics and the consequent rise in antimicrobial resistance (AMR) represent a major global health threat.^1,2^ Although guidance exists for the implementation of national antimicrobial stewardship (AMS) action plans, the development of effective policies depends heavily on the availability of reliable data on antimicrobial prescribing and consumption.^1,2^ Therefore, an analysis of national prescription data can be the building block for gaining valuable insights on the prescribing habits of the clinicians in the community and help identify future targets for tailored AMS interventions, targeting specific populations and prescribing patterns.

Cyprus, an island nation in the midst of a healthcare transition following the introduction of a unified National Health System (NSHS) in 2019, is actively addressing key areas of focus in establishing robust surveillance and stewardship frameworks. As part of this ongoing transformation, the country is working to align its healthcare practices with EU priorities on antimicrobial resistance, infection prevention and control (IPC), and antimicrobial stewardship. Historically, antibiotics in Cyprus were readily accessible without a prescription in the community, a practice that was formally prohibited following the establishment of the NHS in 2019. Despite legal restrictions, several studies documented the continued over-the-counter (OTC) sale of antibiotics by pharmacists, particularly prior to these regulatory reforms.^3,4^ Community pharmacists in Cyprus also exhibited varied levels of awareness and involvement in antimicrobial stewardship, highlighting the need for enhanced training and stricter enforcement of prescription policies. Moreover, public knowledge about antibiotics and antimicrobial resistance remains limited. A survey in Cyprus indicated that nearly one-third of the population mistakenly believed antibiotics were effective against viral infections, and over 70% lack a clear understanding of resistance mechanisms.^4^ These gaps in both provider behavior and public awareness underlined the importance of targeted education and policy enforcement to support AMS efforts in the Cypriot healthcare setting.

Despite the importance of comprehending antibiotic use, the data available from Cyprus has been limited to aggregated antibiotic consumption data reported to the European Surveillance of Antimicrobial Consumption Network (ESAC-Net) of the European Centre for Disease Prevention and Control (ECDC), without differentiation between hospital and community settings.^5–9^ This lack of stratification hampers targeted future intervention efforts and effective national and local policymaking. In addition, Cyprus was ranked first and second in antimicrobial consumption among the 29 European countries in 2020 and 2021.^7^ According to the 2022 ECDC report, the increase in the mean total national consumption of antibacterials for systemic use (community and hospital sector) in Cyprus for the period 2013 – 2022 was 11.4%.^6^ In 2022, Cyprus had the highest total consumption (community and hospital sectors combined) of antibacterials among European countries, measured at a population-weighted mean consumption of 33.5 DDD per 1 000 inhabitants per day. In comparison, the mean consumption in the EU was 19.4.^6^ Notably, the 2023 joint ECDC and WHO report highlighted that Cyprus exhibits one of the highest EU rates of antimicrobial resistance.^10^ Cyprus is currently one of the four countries with *Escherichia coli* fluoroquinolone resistance over 50%, as well as one of the five countries to report *Enterococcus faecium* vancomycin resistance above 50%.^6^ Furthermore, to this date, there is a lack of data on how antibiotics are prescribed in the community in Cyprus according to the AWARE classification.^11^ According to the recent 2022 ECDC (ESAC-Net) report, the total consumption of “Access” group antibacterials in Cyprus (including both hospital and community settings) was 55.4%, falling short of the EU target of 60%.^11,12^

Although community antibiotic use surveillance is an important component in AMS, there has been a lack of information on a national level in Cyprus. To this direction, this study aims to bridge this gap and elucidate the patterns of national antibiotic prescribing from 2020 to 2022 in outpatient services and to identify opportunities for future AMS priorities. The ultimate target is to provide guidance for drafting effective and efficient AMS policies.

## Material and methods

### Community antibiotic consumption data from national health insurance organization database (HIO)

Prescription data were extracted from the National Health Insurance Organization (HIO) for the period 2020 – 2022. Data extracted for each prescription were de-identified, abiding to GDPR legislation, and included the year of prescription, Anatomical Therapeutic Chemical Code (ATC), specialty of the prescriber, and age of the patient. As in ESAC-Net summaries,^6,7^ antibiotics were divided in six main categories according to the ATC level 3 classification: penicillins (J01C), cephalosporins and other beta-lactams (J01D), macrolides, lincosamides, and streptogramins (J01F), tetracyclines (J01A), quinolones (J01M), and sulfonamides and trimethoprim (J01E); rectal metronidazole (P01AB01) was also included.

Additionally, we compared the prescription of broad-spectrum penicillins, cephalosporins, macrolides (except erythromycin) and fluoroquinolone s(J01(CR + DC + DD+(FA–FA01)+MA)) to the consumption of narrow-spectrum penicillins, cephalosporins and erythromycin (J01(CA + CE + CF + DB + FA01).

### Analyses by AWaRe classification

The Access, Watch and Reserve (AWaRe) classification of antimicrobials was used which was developed by World Health Organization (WHO) in 2017 and updated in 2021,^11,12^ to aid in the evaluation and monitoring of antibiotic use and support AMS efforts. The WHO 13th General Programme of Work 2019 – 2023 includes a country-level target of at least 60% of total antibiotic consumption being Access group antibiotics. According to AWaRE the antimicrobials were classified accordingly in three categories: “Access” (best therapeutic value, with low potential for antimicrobial resistance), “Watch” (higher AMR potential and priority targets for stewardship and monitoring efforts), and “Reserved” (restricted to confirmed or suspected infections with multidrug-resistant microorganisms.

***Statistical analysis***

Statistical analyses and data visualization were carried out using the statistical package Stata SE v.18. Prescriptions are presented with absolute and relative frequencies (%) as well as graphically with bar charts. Antibiotic prescribing rates per 1,000 beneficiaries were calculated based on the National official data on beneficiaries each year as indicated by the Health Insurance Organization Cyprus (HIO).^13^ The number of beneficiaries was not constant and increased from 755,878 in 2020 to 916,803 in 2022. The relative percentage change of antibiotic prescribing rates per 1000 beneficiaries between 2020 and 2022 was calculated. Trends of yearly antibiotic prescribing rates were evaluated using simple run charts.

***Bioethics approval***

All data used in this study were fully anonymized and extracted in accordance with applicable national and European Union regulations, including the General Data Protection Regulation (GDPR; Regulation (EU) 2016/679). The Health Insurance Organization (HIO) provided access to de-identified prescription records, and no personal identifiers were included in the data set. Ethical approval for this study was obtained from the Cyprus National Bioethics Committee (Protocol No. EEBK EΠ 2023.01.286).

## Results

***Annual rate of antibiotic prescriptions***

During the study period (2020–2022), 1,458,723 antibacterial prescriptions for systemic use (oral J01 & P01AB01) were ordered in Cyprus for outpatients. The annual rates of prescriptions per 1,000 beneficiaries showed an increasing trend over this period: 535.7 in 2020, 532.4 in 2021, and 653 in 2022, indicating a 21.9% increase from 2020 to 2022.

***Distribution of prescriptions by ATC code***

**Figure 1.**
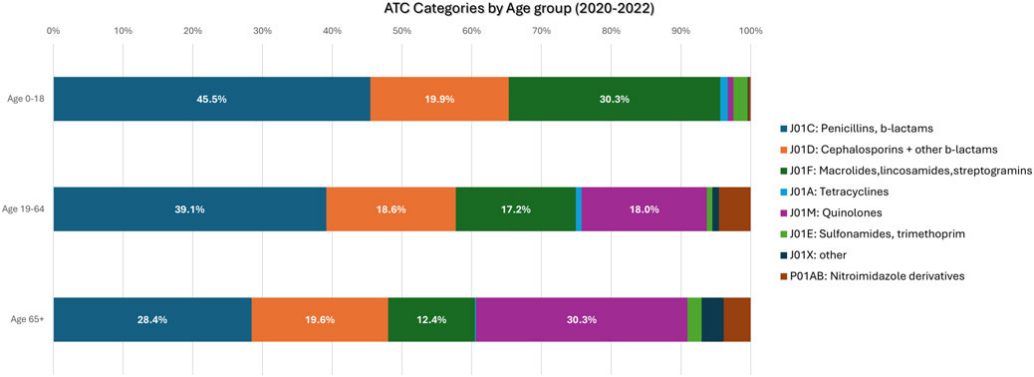
Community antibiotic prescriptions by anatomical therapeutic chemical code (ATC) category and age group in Cyprus, 2020 – 2022.

The distribution of prescriptions by ATC code antibiotic class is depicted in Table 1. Overall, the three most frequently prescribed categories were J01C (Penicillins, b-lactams), J01D (Cephalosporins and other b-lactams) and J01F (Macrolides, lincosamides, streptogramins) with no major difference across the years; almost half (45.5%) of prescribed antibiotics in children are beta-lactam penicillins (J01C) vs 36.1% in adults. Almost similar prescribing rates were recorded in macrolides, whereas quinolones were more frequently prescribed in adults (21.4%) than children (.8%) (Table 1, Figure 1). Over the study period, more than 95% of the antibiotics prescribed in the community were broad-spectrum antibiotics (Table 1).

**Table 1.** Community antibiotic prescription by ATC category and spectrum, total, per 1 000 beneficiaries, by age and by year in Cyprus, 2020 – 2022

			per 1.000 beneficiaries^*^	Children		Adults				By Year						
	Total			Age 0 – 18		Age 19 – 64		Age 65+		2020		2021		2022		2020 – 2022
**Categories**	**N**	**%**		**N**	**%**	**N**	**%**	**N**	**%**	**N**	**%**	**N**	**%**	**N**	**%**	**% change**
J01C: Penicillins, b-lactams	553,961	38.0%	219.17	132,226	45.5%	328,079	39.1%	93,656	28.4%	155,498	38.4%	169,094	37.2%	229,369	38.3%	−.1
J01D: Cephalosporins, other b-lactams	278,252	19.1%	110.09	57,790	19.9%	155,897	18.6%	64,565	19.6%	81,633	20.2%	85,007	18.7%	111,612	18.6%	−1.5
J01F: Macrolides, lincosamides, streptogramins	273,192	18.7%	108.08	88,222	30.3%	143,983	17.2%	40,987	12.4%	68,854	17.0%	82,944	18.2%	121,394	20.3%	3.3
J01A: Tetracyclines	10,904	.7%	4.31	3,169	1.1%	7,235	.9%	500	.2%	3,230	.8%	4,153	.9%	3,521	.6%	−.2
J01M: Quinolones	252,762	17.3%	100.00	2,270	.8%	150,486	18.0%	100,006	30.3%	70,101	17.3%	83,359	18.3%	99,302	16.6%	−.7
J01E: Sulfonamides, trimethoprim	19,270	1.3%	7.62	6,011	2.1%	6,659	.8%	6,600	2.0%	5,310	1.3%	6,648	1.5%	7,312	1.2%	−.1
J01X: Other	18,452	1.3%	7.30	281	.1%	7,743	.9%	10,428	3.2%	4,966	1.2%	6,095	1.3%	7,391	1.2%	.0
P01AB: Nitroimidazole derivatives	51,930	3.6%	20.55	931	.3%	38,219	4.6%	12,780	3.9%	15,353	3.8%	17,829	3.9%	18,748	3.1%	−.7
**Total**	**1,458,723**		**577.12**	**290,900**		**838,301**		**329,522**		**404,945**		**455,129**		**598,649**		
**Antibiotic Spectrum**	**N**	**%**								**N**	**%**	**N**	**%**	**N**	**%**	
Narrow spectrum	50,609	3.9%								15,156	4.2%	16,499	4.1%	18,954	3.5%	−.7
Broad spectrum	1,247,135	96.1%								344,278	95.8%	383,473	95.9%	519,384	96.5%	+.7
**Total**	**1,297,744**									**359,434**		**399,972**		**538,338**		
**Broad / Narrow Ratio**	**24.6**									**22.7**		**23.2**		**27.4**		

***Prescribing patterns according to WHO AWaRe classification***

Over the 3-year period, Cypriot prescriptions in the community were 45.7% “Access,” 54.3% “Watch” and less than .1% Reserve (Table 2). Looking in more detail, the prescription rate of “Access” antibiotics has not substantially changed over the study period but still decreasing from 46.4 in 2020 to 42.3% in 2022 (Table 2, Supplemental Figure 1)

**Table 2.** Community antibiotic prescriptions according to the WHO AWaRe stratification in Cyprus, 2020 – 2022

	Total		2020		2021		2022	
**AWARE classification**	**N**	**%**	**N**	**%**	**N**	**%**	**N**	**%**
Access	665,450	45.7%	187,526	46.4%	207,234	45.6%	270,690	42.3%
Watch	791,734	54.3%	217,045	53.6%	247,348	54.4%	327,341	54.7%
Reserve	306	< .1%	34	< .1%	132	.0%	140	< .1%
**Total**	**1,457,490**		**404,605**		**454,714**		**598,171**	

***Top prescribers***

Overall, most prescriptions are ordered by General Practitioners, Internal Medicine consultants, pediatricians, and obstetricians/gynecologists as shown in Table 3. Internal medicine and pediatrics account for 65% of prescriptions.

**Table 3.** Community antibiotic prescriptions in Cyprus by medical specialty per year in Cyprus, 2020 – 2022

	Total		2020		2021		2022	
**Medical specialty**	**N**	**%**	**N**	**%**	**N**	**%**	**N**	**%**
General/internal medicine	699,189	47.9%	200,528	49.5%	206,180	45.3%	292,481	48.9%
General pediatrics	242,681	16.6%	60,729	15.0%	68,895	15.1%	113,057	18.9%
Obstetrics/gynecology	109,561	7.5%	29,345	7.3%	38,735	8.5%	41,481	6.9%
Urology	72,566	5.0%	21,502	5.3%	24,897	5.5%	26,167	4.4%
Otorhinolaryngology	60,575	4.2%	19,839	4.9%	19,693	4.3%	21,043	3.5%
Dermatology-venereology	55,619	3.8%	18,904	4.7%	20,014	4.4%	16,701	2.8%
General surgery	54,392	3.7%	14,962	3.7%	18,888	4.2%	20,542	3.4%
Orthopedics	33,395	2.3%	7,804	1.9%	11,740	2.6%	13,851	2.3%
Respiratory medicine	25,828	1.8%	7,867	1.9%	8,034	1.8%	9,927	1.7%
Gastroenterology	20,399	1.4%	6,297	1.6%	7,290	1.6%	6,812	1.1%
Other	84,518	5.8%	17,168	4.2%	30,763	6.8%	36,587	6.1%
**Total**	**1,458,723**		**404,945**		**455,129**		**598,649**	

## Discussion

The results of this first national preliminary study on antibiotic prescription trends in Cyprus from 2020 to 2022 indicated a substantial rise in the number of antibacterial prescriptions for systemic use among outpatients. With 1,458,723 prescriptions recorded, the data revealed an increase in the annual prescription rates per 1,000 beneficiaries, escalating from 535.7 in 2020 to 653 in 2022, an increase of 21.9% over two years. High rates of quinolone prescriptions in adults in the community were recorded and the percentage of prescriptions with “Access” antibiotics were below the WHO and EU targets. The top three prescribers identified are internists, pediatricians and obstetrics/gynecology, accounting for 72% of all community antibiotic prescriptions in Cyprus for the study period.

In 2012, Cyprus unveiled a National Strategy to address Antimicrobial Resistance aimed at preserving the effectiveness of antimicrobial therapies for both humans and animals. The strategy underscored the importance of cross-sectoral collaboration involving human medicine, veterinary medicine, livestock, and agriculture fields.^14,15^ The primary goal was to develop and implement measures to reduce AMR in Cyprus, with a focus on surveillance, antibiotic consumption, use of antibiotics in animal husbandry, and the prevalence of hospital-acquired infections. The strategy offered a comprehensive overview of current data to assess the situation in Cyprus and identified necessary areas of improvement.^15^ Since the establishment of a national AMR surveillance system in 2012, the Cypriot National Antibiotics Committee has issued several annual reports analyzing this data. Recently, the Ministry of Health Cyprus announced the development of a new national strategy targeting multidrug-resistant organisms (MDROs), alongside the re-establishment of the national committee for infection control, AMS and AMR.^15^ This new national strategy will incorporate a formal antimicrobial stewardship program (ASP), targeted training framework, and antimicrobial stewardship groups within hospitals. In addition, the HIO has recently initiated a national program with patient safety and quality indicators including the consumption monitoring of specific broad-spectrum antibiotics.^13^ Nevertheless, Cyprus has yet to develop and implement a national AMS guideline, leading many clinicians till now to prescribe empirical antimicrobial therapies based on individual judgment. ^16^

According to our study findings, while a direct comparison between prescriptions per 1,000 beneficiaries and the ECDC-DDD per 1,000 inhabitants was not feasible, we analyzed the distribution of prescribed ATC classes relative to their reported consumption. In Cyprus, beta-lactams and penicillins (J01C) accounted for 38% of all prescriptions, which is lower than the EU/EEA average of 44.9%. In contrast, quinolones (J01M) represented 17% of prescriptions, significantly exceeding the EU average of 7.3%. Given that *Escherichia coli* fluoroquinolone resistance in Cyprus surpasses 50%, the high prescribing rate of quinolones (J01M) requires further scrutiny. Another key discrepancy was observed in the use of tetracyclines (J01A), which constituted 9% of prescriptions in the EU/EEA but only .7% in Cyprus. This difference may reflect a higher prevalence of conditions requiring alternative treatments or highlight a potential area for targeted antimicrobial stewardship interventions. The use of macrolides, lincosamides, and streptogramins (J01F) in Cyprus (18.7%) was largely comparable to the EU/EEA average of 17.4%. Notably, beta-lactam penicillins (J01C) were the most frequently prescribed antibiotics in children, comprising nearly half of all pediatric prescriptions, a trend that aligns with global pediatric prescribing patterns. Previous research has already demonstrated substantial variability in outpatient antibiotic prescribing practices across European countries, further emphasizing the need for context-specific AMS strategies.^16–22^ These findings align also with previous research indicating significantly lower outpatient antibiotic use in Northern compared to Southern European countries.^17^

In Cyprus the predominance of “Watch” category antibiotics (54.3%) over “Access” antibiotics (45.7%) raised concerns, as “Watch” antibiotics carry a higher risk of promoting antimicrobial resistance. When assessing antibacterial use based on the WHO’s AWaRe classification, our findings cannot be directly compared with the ESAC-Net Report. This is because the report provides only aggregated consumption data for both hospitals and communities, and the available datasets lack specific details on AWaRe consumption within the community for the ATC J01 group. Notably, the ESAC-Net Report states that in 2022, over 50% of Cyprus’s total antibiotic consumption fell within the “Access” category, whereas our study estimated for the community consumption in the same year were lower at 42.3%. This Figure falls well below both the WHO’s recommended target of 60% and the EU’s target of 70% for “Access” antibiotic consumption.^11,12^

While this study provides valuable initial insights into antibiotic prescribing patterns in the community of Cyprus, several limitations must be acknowledged. First, our analysis was based on nationwide prescription data rather than DDDs, which limited direct comparability with other antimicrobial consumption reports that rely on DDD metrics. However, as the primary objective was to assess prescribing patterns, the number of prescriptions served as a more appropriate indicator for evaluating physician behavior. An important consideration was that unit-dose dispensing was not implemented in Cyprus, meaning antibiotics are dispensed in fixed package sizes that may not always align with the recommended treatment durations in national guidelines. This discrepancy could lead to an overestimation of antibiotic consumption when using DDDs. In this regard, analyzing prescription data, as done in this study, offers an alternative perspective that may more accurately reflect community prescribing practices within the Cypriot healthcare system. Even though the observation period (2020 – 2022) is relatively short and overlaps with the COVID-19 pandemic—a significant contextual factor likely influencing prescribing behaviors—we emphasize that these are the actual national data from that time and thus reflect real-world trends irrespective of underlying causes.

Additionally, our study focused solely on quantitative data, without exploring the underlying reasons for prescribing behaviors or the potential influence of external factors, such as public health campaigns, antimicrobial stewardship initiatives, or seasonal variations in infections. The available data set would not allow to link prescriptions with clinical outcomes or to account for potential confounding factors like appropriateness, healthcare accessibility, physician prescribing habits, and patient adherence. Assessing these factors in future studies could provide further context to the observed trends and help inform targeted interventions.

This study does not allow for direct correlations of antibiotic consumption patterns with antimicrobial resistance trends in Cyprus. Although AMR data are collected by the Ministry of Health, they are not yet linked with outpatient prescribing data maintained by the Health Insurance Organization (HIO). This reflects a broader opportunity for improvement in developing an integrated national database that connects antibiotic use with resistance outcomes. While our study offers valuable initial insights into community prescribing practices, we would like to underline the importance of establishing a unified, cross-sectoral monitoring framework. Such a system would support a more comprehensive evaluation of the relationship between antibiotic use and resistance trends and would strengthen ongoing national antimicrobial stewardship initiatives.

The study’s strengths lied in its provision of the first comprehensive national data on outpatient antibiotic prescription trends in Cyprus. Utilizing a large data set over an extended period allowed for robust analysis and reliable identification of initial trends. This study contributed significantly to the ongoing surveillance of antibiotic use, providing healthcare professionals and policymakers with valuable preliminary insights to audit outpatient prescribing practices. These findings can help identify initial future key areas for improvement, with the overarching goal of enhancing national antibiotic stewardship efforts.

In conclusion, this study offered a detailed overview of community antibiotic prescribing patterns at the national level. The observed increasing prescription rates, high prevalence of quinolone prescriptions, and low rates of “Access” antibiotics emphasize the urgent need for strengthened antibiotic stewardship programs in Cyprus. Future research should focus on investigating the underlying factors driving these prescribing patterns and assessing the effectiveness of policy interventions aimed at reducing antibiotic use and mitigating resistance trends. This preliminary data set offers an unprecedented opportunity to assess antibiotic prescribing trends, pinpoint areas requiring intervention, and provide invaluable insights to inform evidence-based antimicrobial stewardship strategies.

## Supporting information

10.1017/ash.2025.10079.sm001 Mitsoura et al. supplementary material 1 Mitsoura et al. supplementary material

10.1017/ash.2025.10079.sm002 Mitsoura et al. supplementary material 2 Mitsoura et al. supplementary material

## Supplementary material

The supplementary material for this article can be found at https://doi.org/10.1017/ash.2025.10079.

## References

[1] O’Neill J. Tackling Drug-Resistant Infections Globally: Final Report and Recommendations 2016, chrome-extension://efaidnbmnnnibpcajpcglclefindmkaj. https://amr-review.org/sites/default/files/160518_Final%20paper_with%20cover.pdf. Published 2016. Accessed February 16, 2025.

[2] Anderson M , Clift C , Schulze K et al. Averting the AMR crisis: what are the avenues for policy action for countries in Europe? Copenhagen (Denmark): European Observatory on Health Systems and Policies; 2019. 31287637

[3] Sullman MJM , Lajunen TJ , Baddal B , Apostolou M. Antibiotics knowledge, attitudes and behaviours among the population living in Cyprus. Antibiotic 2023;12:897. 10.3390/antibiotics12050897 PMC10215266 37237800

[4] Michaelidou M , Karageorgos SA , Tsioutis C. Antibiotic use and antibiotic resistance: public awareness survey in the republic of Cyprus. Antibiotic 2020;9:759. 10.3390/antibiotics9110759 PMC7692346 33143207

[5] 2021 TrACSS Country Report on the Implementation of National Action Plan on Antimicrobial Resistance (AMR). Website. https://cdn.who.int/media/docs/default-source/antimicrobial-resistance/amr-spc-npm/tracss/tracss-2021-cyprus.pdf. Published 2021. Accessed February 16, 2025.

[6] European Centre for Disease Prevention and Control, Antimicrobial Consumption in the EU/EEA (ESAC-Net) Annual Epidemiological Report 2022. ECDC. https://www.ecdc.europa.eu/en/publications-data/surveillance-antimicrobial-consumption-europe-2022. Published 2023. Accessed February 16, 2025

[7] European Centre for Disease Prevention and Control (ECDC). Antimicrobial Consumption in the EU/EEA (ESAC-Net)—Annual Epidemiological Report 2021. Stockholm, Sweden. website. https://www.ecdc.europa.eu/en/publications-data/surveillance-antimicrobial-consumption-europe-2021. Published 2022. Accessed February 16, 2025.

[8] European Centre for Disease Prevention and Control. website. Antimicrobial Resistance Surveillance in Europe 2022: 2020 Data. https://www.ecdc.europa.eu/en/publications-data/antimicrobial-resistance-surveillance-europe-2022-2020-data. Published 2022. Accessed February 16, 2025.

[9] Mitsoura E , Kopsidas I , Charalambous P , et al. Community antibiotic consumption in Cyprus for the Period 2015 to 2022. Antibiotics (Basel) 2024;13:52. 38247611 10.3390/antibiotics13010052 PMC10812799

[10] European Centre for Disease Prevention and Control (ECDC) & World Health Organization Regional Office for Europe (WHO/Europe): 2023. Antimicrobial resistance surveillance in Europe 2023 – 2021 data. ECDC. https://www.ecdc.europa.eu/sites/default/files/documents/Antimicrobial%20resistance%20surveillance%20in%20Europe%202023%20-%202021%20data.pdf. (Accessed: 30 May 2025).

[11] World Health Organization, WHO Access, Watch, Reserve (AWaRe) Classification of Antibiotics for Evaluation and Monitoring of Use, 2021. World Health Organization. Website. https://www.who.int/publications/i/item/2021-aware-classification. Published 2021. Accessed February 16, 2025.

[12] World Health Organization. Thirteenth general programme of work, 2019–2023: promote health, keep the world safe, serve the vulnerable. Geneva World Health Organization. Website. https://www.who.int/publications/i/item/thirteenth-general-programme-of-work-2019-2023.Published. Published 2019. Accessed February 16, 2025.

[13] Heath Insurance Organization Cyprus. https://www.gesy.org.cy/sites/Sites?d=Desktop&locale=en_US&lookuphost=/en-us/&lookuppage=hioroleandresponsibilities. Published 2018. Accessed February 16, 2025.

[14] WHO National Action Plans and Monitoring and Evaluation (NPM). Cyprus: National action plan on antimicrobial resistance (Greek). Website. https://www.who.int/publications/m/item/cyprus-national-action-plan-on-antimicrobial-resistance https://www.gov.cy/moh/. Published 2012. Accessed February 16, 2025..

[15] Spernovasilis N , Tsioutis C. Not surprising: a rebound in antibacterial consumption in Europe, with Cyprus and Greece on the podium. J AntimicrobChemother 2024;79:933–934. 10.1093/jac/dkae055 PMC11062937 38442334

[16] Goossens H , Ferech M , Vander Stichele R , Elseviers M. Outpatient antibiotic use in Europe and association with resistance: a cross-national database study. Lancet 2005;365:579–587. 15708101 10.1016/S0140-6736(05)17907-0

[17] Outpatient Antibiotic Prescriptions — United States, 2022, CDC. Website. https://www.cdc.gov/antibiotic-use/data/report-2022.html. Published 2022. Accessed February 16, 2025.

[18] Clavenna A , Bonati M. Differences in antibiotic prescribing in paediatric outpatients. Arch Dis Child 2011;96:590–595. 21233078 10.1136/adc.2010.183541

[19] Holstiege J , Schink T , Molokhia M , et al. Systemic antibiotic prescribing to paediatric outpatients in 5 European countries: a population-based cohort study. BMC Pediatr 2014;14:174. 24997585 10.1186/1471-2431-14-174 PMC4099488

[20] Shah M , Barbosa TM , Stack G , Fleming A. Trends in antibiotic prescribing in primary care out-of-hours doctors’ services in Ireland. JAC Antimicrob Resist 2024;6:dlae009. 38343625 10.1093/jacamr/dlae009 PMC10854216

[21] Treibich C , Lescher S , Sagaon-Teyssier L , Ventelou B. The expected and unexpected benefits of dispensing the exact number of pills. PLoS ONE 2017;12:e0184420. 28926636 10.1371/journal.pone.0184420 PMC5604959

[22] Holstiege J , Schulz M , Akmatov MK , Steffen A , Bätzing J. Marked reductions in outpatient antibiotic prescriptions for children and adolescents - a population-based study covering 83% of the paediatric population, Germany, 2010 to 2018. Euro Surveill 2020; 25:1900599. 32762794 10.2807/1560-7917.ES.2020.25.31.1900599 PMC7459269

