# Supplementary figures and images for: National outpatient antibiotic prescriptions in Cyprus, 2020–2022
Source: Antimicrob Steward Healthc Epidemiol. 2025 Oct 15;5(1):e271. doi: 10.1017/ash.2025.10079 (PMC12616571; doi:10.1017/ash.2025.10079)

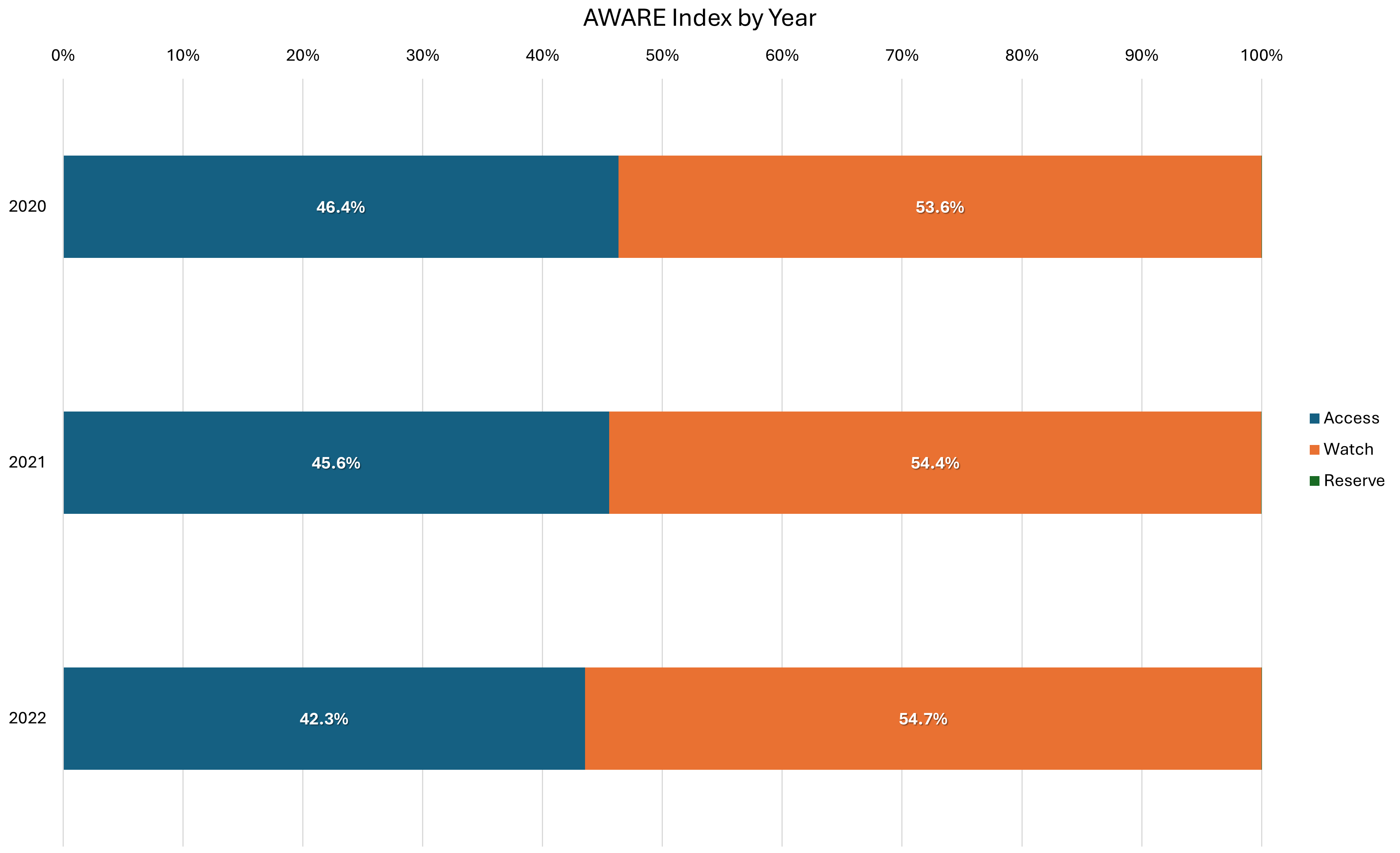

Supplement: Mitsoura et al. supplementary material 1 — Mitsoura et al. supplementary material [file S2732494X2510079Xsup001.tiff]
