# Supplementary material for: National outpatient antibiotic prescriptions in Cyprus, 2020–2022
Source: Antimicrob Steward Healthc Epidemiol. 2025 Oct 15;5(1):e271. doi: 10.1017/ash.2025.10079 (PMC12616571; doi:10.1017/ash.2025.10079)
Supplement: Mitsoura et al. supplementary material 2 — Mitsoura et al. supplementary material [file S2732494X2510079Xsup002.docx]

**Supplemental Table 1. Number of prescriptions and rate of prescriptions per 1000 beneficiaries per year, 2020-2022**

|  | **Total Number of Prescriptions (Oral J01 + P01AB)** | **Rate of Prescriptions per 1.000 Beneficiaries**  **(Oral J01 + P01AB)** |
| --- | --- | --- |
| 2020 | 404,945 | 535.7 |
| 2021 | 455,129 | 532.4 |
| 2022 | 598,649 | 653.0 |
| **TOTAL** | **1,619,375** | **534.2** |
